# Editorial Head and Neck Radiology, Annual Meeting of the Belgian Society of Radiology (BSR) 17th November 2018

**DOI:** 10.5334/jbsr.1663

**Published:** 2018-11-17

**Authors:** Robert Hermans, Jacques Widelec

**Affiliations:** 1 UZ Leuven, BE; 2 HIS-IZZ, BE

One of the themes during this Annual Meeting is the field of Head and Neck Radiology. During two sessions, one organized by the Young Radiologists Section, lectures will be presented on different topics of interest both to the general and more specialized radiologists.

This editorial presents the lectures and topics during the session moderated by Dr. Yannick De Brucker (UZ Brussel, VUB) and Dr. Jacques Widelec (HIS-IZZ Bruxelles).

**Figure:**
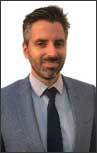
Dr. Yannick De Brucker

**Figure:**
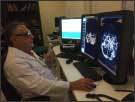
Dr. Jacques Widelec


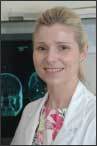
 The first lecture is presented by Dr. Anja Bernaerts (St. Augustinus Hospital GZA, Antwerpen). Dr. Bernaerts graduated as an MD from the University of Antwerp in 2000. She completed her radiology residency at the Sint-Maarten hospital in Duffel, the Gasthuiszusters Antwerp (GZA) hospital Sint-Augustinus in Antwerp, and the University hospital Antwerp in 2005. She currently works as a staff member of the department of radiology of the GZA hospitals, Antwerp, Belgium, with breast radiology and head and neck radiology sub-specializations. In 2013 she obtained the European Diploma in Breast Imaging and in 2015 she also obtained the European Diploma in Head and Neck Radiology.

She is a team member of the Breast Unit of the GZA Hospitals, which has been certified with a European Cancer Care Certification. In performing Head and Neck radiology exams, she works closely together with Dr. Bert De Foer and Prof. Dr. Jan W. Casselman.

In the field of head and neck radiology, she also has a particular expertise in dental and maxillo-facial imaging, disposing also of the Certificate of Competence in the Use of Cone Beam CT in the Dental Practice, obtained at the Catholic University of Leuven in 2013.

She authored and/or co-authored 30 peer reviewed articles and five chapters in books.

She is also the principal investigator of the in-progress study of the department of Radiology and the European institute for ORL-HNS at the GZA hospitals on the value of MRI in Menière’s Disease.

During her lecture ‘**MRI in Ménière’s Disease**’, she will show the possibilities of this relatively new MR technique in the management of patients with otological symptoms possibly caused by endolymphatic hydrops.


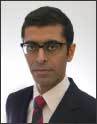
 The second lecture is presented by Dr. Kunwar Bhatia. Dr. Bhatia is consultant radiologist, with a special focus on head and neck radiology, working in the Imperial College Healthcare NHS Trust, Division of Imaging and Interventional Radiology, London, UK. He previously had an academic tenure as Clinical Associate Professor (Radiology) in The Chinese University of Hong Kong (2007–2016).

In this session he will address ‘**Imaging of Salivary Masses**’. There are numerous causes of salivary enlargement, which can be broadly divided into focal masses, which may be neoplastic or non-neoplastic, and diffuse salivary diseases, which may be secondary to obstruction of salivary ducts (obstructive sialadenitis) or a wide range of other infective, inflammatory, autoimmune and other pathologies. Imaging fulfills several roles in the initial work-up including localization, diagnosis or characterization, staging of malignancies, image guided biopsy and therapeutic interventions (aspirations and stone extractions).


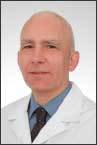
 The third lecture is presented by Prof. Dr. Robert Hermans (UZ Leuven, KUL). Prof. Hermans is staff member in the department of radiology in the UZ Leuven since 1992. His primary interest is head and neck radiology. He served as president of the International Cancer Imaging Society (ICIS). He is also past president of the Royal Belgian Society of Radiology (RBSR). From 2010 till 2017, he served as editor-in-chief of Insights into Imaging, journal of the European Society of Radiology (ESR). Dr. Robert Hermans authored about 180 articles and 40 book chapters, mainly on head and neck imaging, with a special focus on cancer imaging. He was editor of four books on this topic.

His lecture deals with ‘**Imaging of Perineural Tumor Spread**’. As there are many nerves present in the head and neck region, these structures may provide tumours the opportunity to spread over a considerable distance from their point of origin. Perineural tumour spread may occur in all head and neck malignancies; adenoid cystic carcinoma, a tumour of salivary gland origin, is notorious for its tendency to spread along nerves. Perineural tumour spread is associated with a decreased survival rate. Imaging diagnosis is important to map the full tumour extent and to avoid tumour progression from unrecognized perineural spread.

